# Characteristics and Outcomes of Anti-interferon Gamma Antibody-Associated Adult Onset Immunodeficiency

**DOI:** 10.1007/s10875-023-01537-0

**Published:** 2023-06-26

**Authors:** Bingqing Zhang, Junpin Fan, Chengjing Huang, Hongwei Fan, Jialin Chen, Xiaoming Huang, Xuejun Zeng

**Affiliations:** 1 Department of General Internal Medicine, State Key Laboratory of Complex Severe and Rare Diseases, Peking Union Medical College Hospital, Chinese Academy of Medical Science & Peking Union Medical College, NO.1 Shuaifuyuan, Dongcheng District, Beijing, 100730 China; 2 Department of Pulmonary, Peking Union Medical College Hospital, Chinese Academy of Medical Science & Peking Union Medical College, NO.1 Shuaifuyuan, Dongcheng District, Beijing, 100730 China; 3 Department of Infectious Disease, Peking Union Medical College Hospital, Chinese Academy of Medical Science & Peking Union Medical College, NO.1 Shuaifuyuan, Dongcheng District, Beijing, 100730 China

**Keywords:** Anti-interferon gamma antibody, Acquired immunodeficiency syndrome, Nontuberculosis mycobacterium infections, Talaromyces, Rituximab

## Abstract

**Purpose:**

Anti-interferon gamma antibody (AIGA) is a rare cause of adult onset immunodeficiency, leading to severe disseminated opportunistic infections with varying outcomes. We aimed to summarize the disease characteristics and to explore factors associated with disease outcome.

**Methods:**

A systematic literature review of AIGA associated disease was conducted. Serum-positive cases with detailed clinical presentations, treatment protocols, and outcomes were included. The patients were categorized into controlled and uncontrolled groups based on their documented clinical outcome. Factors associated with disease outcome were analyzed with logistic regression models.

**Results:**

A total of 195 AIGA patients were retrospectively analyzed, with 119(61.0%) having controlled disease and 76 (39.0%) having uncontrolled disease. The median time to diagnosis and disease course were 12 months and 28 months, respectively. A total of 358 pathogens have been reported with nontubercular mycobacterium (NTM) and *Talaromyces marneffei* as the most common pathogens. The recurrence rate was as high as 56.0%. The effective rates of antibiotics alone, antibiotics with rituximab, and antibiotics with cyclophosphamide were 40.5%, 73.5%, and 75%, respectively. In the multivariate logistic analysis, skin involvement, NTM infection, and recurrent infections remained significantly associated with disease control, with ORs of 3.25 (95% CI 1.187 ~ 8.909, *P* value = 0.022), 4.74 (95% CI 1.300 ~ 17.30, *P* value = 0.018), and 0.22 (95% CI 0.086 ~ 0.551, *P* value = 0.001), respectively. The patients with disease control had significant AIGA titer reduction.

**Conclusions:**

AIGA could cause severe opportunistic infections with unsatisfactory control, particularly in patients with recurrent infections. Efforts should be made to closely monitor the disease and regulate the immune system.

**Supplementary Information:**

The online version contains supplementary material available at 10.1007/s10875-023-01537-0.

## Introduction

Interferon gamma (IFNγ), the only type II IFN, plays an important role in the immunity against intracellular bacteria, as genetic defects in its signaling pathway would lead to genetic susceptibility to opportunistic infections, such as infections with mycobacteria, *Talaromyces marneffei* (TM) and Salmonella. In 2004, Hoflich et al. [1] reported a 25-year-old Thai female with disseminated *Mycobacterium cheloneae* and *Burkholderia cocovenenans* infection. Peripheral blood monocytes (PBMCs) from the patient revealed normal IFNγ production but defective IFNγ activity. Further analysis demonstrated that plasma IgG depletion removed the anti-IFNγ activity, suggesting the existence of a neutralizing autoantibody against IFNγ [1]. Doffinger et al. [2] reported a 47-year-old Filipino male with multiple disseminated infections who was negative for known Mendelian defects in the IL12/INFγ pathway. His serum showed an intact response to the purified-protein derivative but defective secretion of IFN-gamma in vitro. They identified a high titer of IgG antibody to IFNγ, which was capable of inhibiting the IFNγ-dependent augmentation of LPS-induced TNFα production [2]. Functional studies showed that the antibody could target a major epitope on free IFNγ that is crucial for IFNγ receptor (IFN-γR) activation [3], thereby inhibiting IFNγ induced pSTAT-1 phosphorylation and cytokine production [4]. Furthermore, the autoantibodies also impair CD4 + Th1 and CD8 + T cell responses [5]. Thus, patients had impaired intracellular immunity, albeit with normal lymphocytes and cellular responses [2, 6].

The development of anti-IFNγ antibody (AIGA) is still unresolved. Wu et al. [7] investigated the serial serums of one patient collected in the past 7 years and found that the antibody appeared 18 months before the clinical onset, suggesting that the antibody was acquired. Lin et al. [3] found a crucial sequence on the antibody with high homology and cross-reactivity to a portion of the Noc2 protein of Aspergillus spp. Rats immunized with Aspergillus Noc2 developed antibodies that reacted with human IFNγ [3]. These results supported a molecular-mimicry model for antibody development. On the other hand, this disease has been mostly reported in Southeast Asia; and even patients reported in the USA were mostly (91%) Asian immigrants [8], suggesting a genetic propensity for the disease. Genetic studies have found a high LD association between several type II HLA alleles with anti-IFNγ antibodies, including DRB1*16:02, DRB1*15:02, DQB1*05:02, and DQB1*05:01 [9, 10], and these risk alleles have synergistic effects in contributing to the disease [9].

Clinically, patients with AIGA-associated adult onset immunodeficiency (AOID) were initially diagnosed with opportunistic infections; whereas, some patients were misdiagnosed with TAFRO syndrome [11], Rosai-Dorfman disease [12], SAPHO syndrome [13], angioimmunoblastic T cell lymphoma [14], or IgG4-related disease [15]. Antibiotic treatments were the first line treatments, although the clinical outcomes varied significantly. Some patients could successfully stop antibiotics with sustained clinical stability, while others deteriorated even with intensive antibiotic treatment [8, 16]. Although several single-center cohort studies have described its clinical presentations, no standardized treatment protocols have been established and prognostic factors of disease outcome are largely unknown. Given the heterogeneity and relative rarity of the disease, large cohort studies on treatment protocols and disease outcomes are lacking. Therefore, we summarize the clinical characteristics of previously reported diseases, aiming to identify the factors associated with disease outcome.

## Material and Methods

### Literature Review

We searched MEDLINE and EMBASE using the terms “anti interferon” OR “anti-interferon” OR “interferon antibody” OR “interferon autoantibody” limited to the English language by using Google Chrome (Google LLC.90.0) up to November 4^th^, 2021. We also searched the Chinese journal database using the same search terms. Publications of other diseases, basic studies on the antibody, reviews, and case series without individual cases were excluded. Cases reported in different publications were included only once. One unreported case from our center was included in the analysis. A manual search from November 1st, 2021, to Dec 1st, 2022, was added to the final cohort.

Demographic data, symptoms, laboratory results, imaging, pathology, treatments, and prognosis were collected according to the case report. Missing data were defaulted as none or unknown. The inclusion criteria were cases with confirmed antibody testing and clinical infections. The exclusion criteria were cases without detailed clinical information, treatment protocol or documented disease outcome.

### Definition and Subgroups

The diagnosis time was the time from symptom onset to the identification of the antibody. The disease time was the time from symptom onset to the reported end of follow-up. Number of pathogens was the number of pathogens the patient had during the disease course. Recurrent infections by the same pathogen in one patient were recognized as one pathogen. The number of infection episodes was the cumulative number of episodes of infection during the disease course. Coinfection of different pathogens during the same time in one patient were recognized as one episode. Patients with more than one infection episode were considered to have recurrent infections. No. of symptoms were the cumulative number of the systems they had suffered, including constitutional symptoms (fever, weight loss, fatigue), skin involvement (erythema, rashes, nodules, pustulosis, and symptoms consistent with Sweet syndrome), bone involvement (bone pain and having imaging evidence of bone involvement), lung involvement (patients with pulmonary symptoms or imaging abnormalities), abscess formation (skin and muscle abscess, deep abscess and fistula formation), bloodstream infection, and bone marrow infection. The patients with no progressive symptoms or infections with or without therapy were defined as the controlled group, while the patients with progressive symptoms or infections or even death were defined as the uncontrolled group.

### Statistical Analysis

Continuous variables are reported as the median (range) and were compared with the Wilcoxon rank sum test. Counting variables were described as numbers (frequencies) and were compared with the chi-square test. Single and multivariate logistic regression were performed to evaluate factors associated with disease control. SPSS and STATA (14.0, Texas USA) were used as statistical tools, and a 2-sided *P* value of less than 0.05 was designated as significant.

## Results

### Demographic Data

**Fig. 1:**
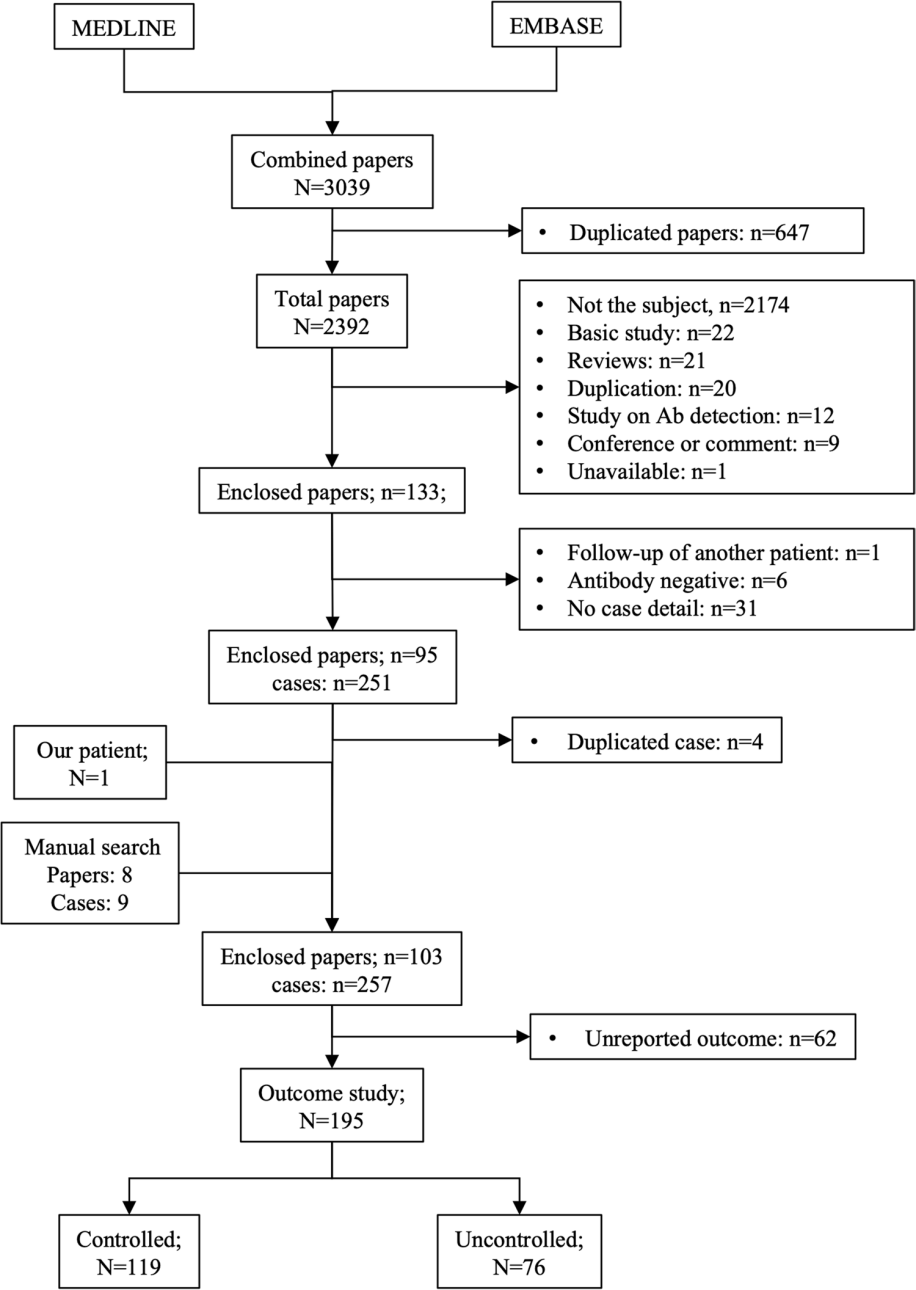
Flow chat of literature review process

A total of 3039 papers were searched during the original search. A total of 2944 papers were excluded due to duplication, not the subject or without detailed clinical information. One unreported case from our center and 9 cases found during a manual search were added. An additional 4 cases were excluded due to duplication, and 62 cases were excluded due to unreported outcomes (Fig. 1, Supplementary Table 1).

**Fig. 2:**
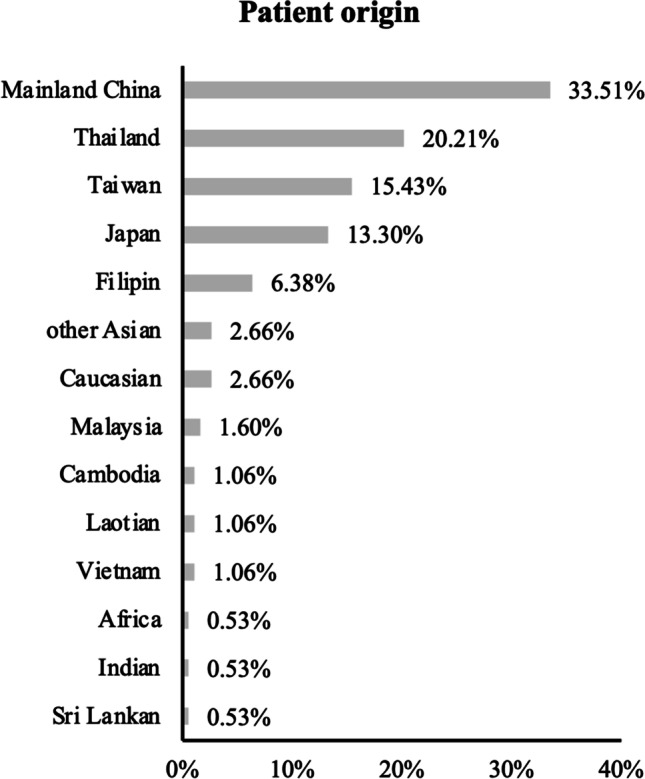
Origins of the reviewed patients. Other Asian refer to patients with Asian background but no detailed information about their country

A total of 195 patients were analyzed in the study, including 101 (51.8%) males and 94 (48.2%) females. The median age at presentation was 55 years old (range 10–87 years). Most patients were from Southeast Asia, including 33.7% (63/189) from mainland China, 20.3% (38/189) from Thailand, 15.5% (29/189) from Taiwan area, and 13.4% (25/189) from Japan. Five (2.7%) patients were of European background and 1 (0.53%) patient was of African background (Fig. 2).

The median time to diagnosis was 12 months (0.7 ~ 612 months). Lymphadenopathy was the most common symptom (145/195, 74.4%), followed by constitutional symptoms (138/195, 70.8%) and pulmonary symptoms (131/195, 67.5%). Other infection loci included bone (90/195, 46.4%), skin (71/195, 36.6%), abscesses (44/195, 22.6%), blood (43/195, 22.2%), and bone marrow (20/195, 10.3%). Twenty-nine patients had documented AIGA titers and reference levels. The median AIGA titer was 79.46 times higher than the upper reference range (range 1.14 ~ 24,000). Twenty-four patients had antibody titer changes recorded during treatment. The median titer after antibiotic treatment alone was 22.9% (range 0.1 ~ 100%) of the baseline titer.

**Fig. 3:**
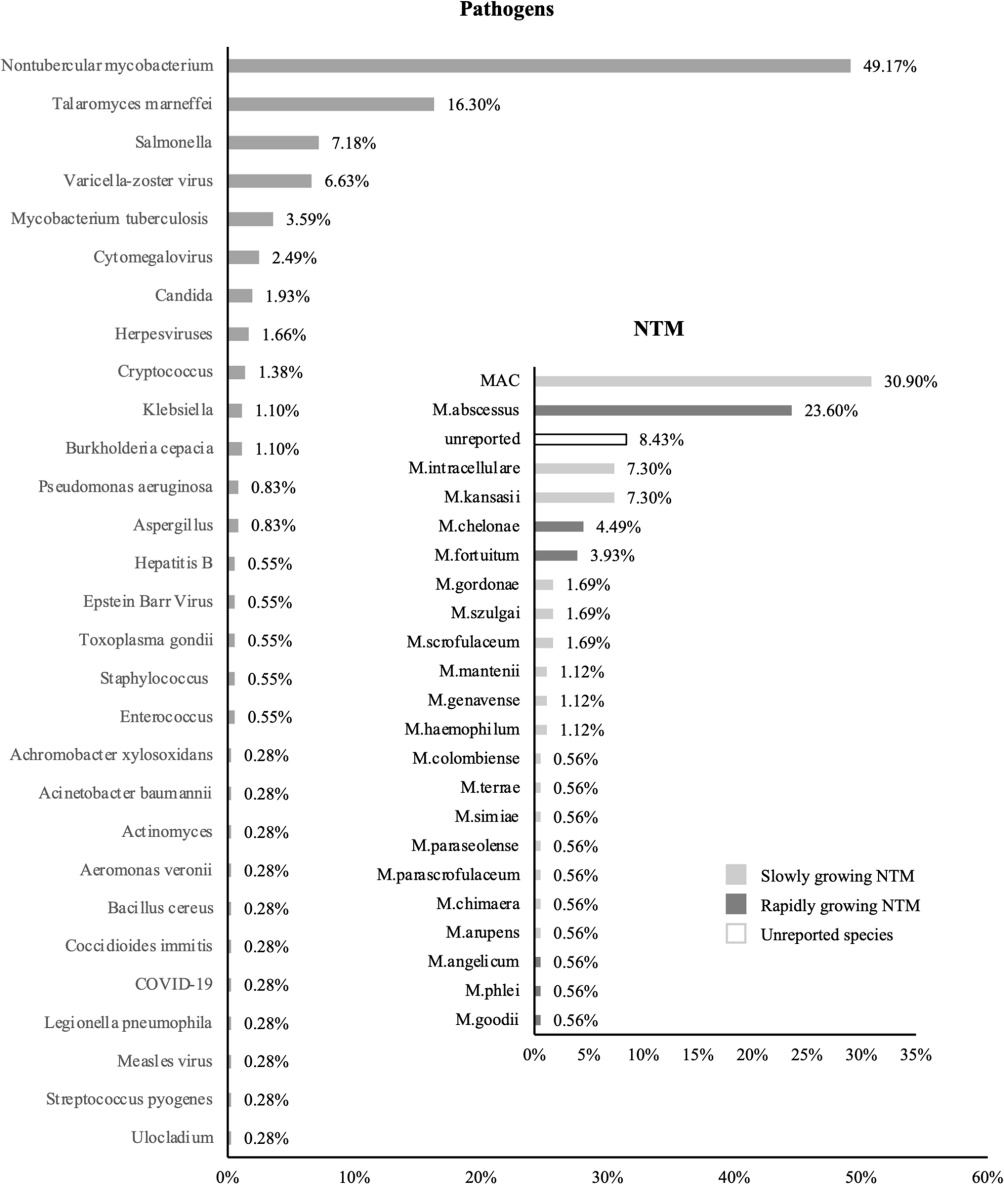
Pathogens reported by the reviewed patients. A total of 362 pathogens have been reported, including 178 non-tuberculosis mycobacterium. The frequency of each pathogen induced infection was labeled in the figure

A total of 362 pathogens have been reported. Nontubercular mycobacterium (NTM) was the most common pathogen (178/362, 49.2%), reported in 77.4% (151/195) of the patients (Fig. 3). Both rapidly growing NTM and slowly growing NTM were reported, with *M. abscessus* (42/178, 23.6%) and *Mycobacterium avium complex* (MAC) (55/178, 30.9%) being the most common pathogens. *Talaromyces marneffei* (TM) was the next most common pathogen (59/362, 16.3%). Other pathogens included Salmonella (26/362, 7.2%), *Varicella zoster* (24/362, 6.6%), and *Mycobacterium tuberculosis* (TB) (13/362, 3.6%) (Fig. 3). 98/195 (50.2%) of the patients had more than one pathogen infection during their disease course, and 78/131 (59.5%) of the patients had more than one infection episode. The median number of infection episodes was 2 (1 ~ 9), and the recurrence rate was 56.0% (79/141). The median time of two infection episodes was 15 months (range 2 ~ 48 months).

### Treatment

The median treatment time was 17 months (range 0.5 ~ 156 months). Antibiotics were the first-line therapy. The patients used a median of 4 (range 1–18) kinds of antibiotics. The reported effective rate of antibiotics alone was 40.5% (79/195).

Thirty-eight (19.5%) patients reported using rituximab (RTX) during their disease course. Most patients started RTX after antibiotics alone failed to control the disease, and the median time to add RTX was 10.9 months (range 30 ~ 164 months) after antibiotic treatment. One patient used RTX before the diagnosis of AOID due to suspected IgG4RD [17], and another patient used RTX due to the diagnosis of diffuse large B cell lymphoma after 2 years [18]. The commonly used protocols were 375 mg/m^2^ weekly to monthly [19–21] or 1 g monthly [22–24]. The median treatment times for the patients with RTX and without RTX were 22 months (1.5 ~ 156 months) and 16 months (0.5 ~ 92 months), respectively. The median antibody titer after RTX treatment was 64.8% (range 49.9 ~ 81.9%) of the baseline titer. After excluding 3 patients without documented RTX effects, and one patient receiving RTX before the diagnosis [17], the general effective rate of RTX was 73.5% (25/34). Four patients had only partial remission [14, 21, 25], and another three patients had progressive infection even after RTX treatment [21, 26]. Two patients failed RTX but achieved disease control with bortezomib [22] or daratumumab [27].

Twenty (10.26%) patients received cyclophosphamide (CTX) after antibiotic failure, and one patient received CTX before the diagnosis of AOID due to suspected IgG4RD [17]. The medium time to add CTX was 19 months (range 19 ~ 175 months) after antibiotic treatment. Laisuan et al. [21] used a dose-escalating protocol, while Chetchotisakd et al. [28] used a fixed dose protocol along with prednisolone. The patients received an average cumulative dose of 5390 mg during an average of 11.2 cycles. The median treatment times of the patients with CTX and without CTX were 39.6 months (7.47 ~ 92 months) and 13.1 months (0.5 ~ 156 months), respectively. The median antibody titer after CTX treatment was 3.75% (range 0.5 ~ 25%) of the baseline titer. The general effective rate was 75% (15/20). Four patients continued to have progressive infections after CTX treatment [21, 28], and 2 of them even failed further RTX treatment [21]. One patient died 3 months later [28].

In addition, 5 (2.6%) patients received IFNγ supplementation [29–32], and 2 of them achieved clinical improvement [29, 30]. One patient only had temporary clinical improvement [31]. The other two patients had no improvement in his immunodeficiency [2, 32]. Thirty-three (16.9%) patients used glucocorticoids. Ten patients used glucocorticoids along with anti-NTM therapy to ease systemic inflammation, and 9 patients used glucocorticoids in addition to RTX or CTX. Eight patients had glucocorticoids for skin or other immunological symptoms, and another 6 for other suspected diseases, such as TAFRO syndrome, IgG4 related disease, SAPHO syndrome, and hematological diseases.

### Outcome

The median follow-up time and general disease course were 16 months (0.3 ~ 96 months) and 28 months (1.6–279 months), respectively. A total of 119 patients had achieved stable disease (controlled group, 61.0%), and 76 patients still had progressive disease or died (uncontrolled group, 39.0%). Twenty-nine (14.8%) patients died. Twenty-six of them died from persistent infection, 2 patients died from both infection and lung adenocarcinoma [33], and another patient died from intracranial hemorrhage [9].

Both patients with and without disease control had similar geographic characteristics and clinical symptoms (Table 1), while the patients with controlled disease had relatively more infection sites, more frequent skin involvement, and fewer comorbidities. In addition, the patients with controlled disease had fewer infection episodes and lower rates of recurrence. The patients with additional RTX and CTX treatment had significantly better disease control, although CTX treatment did not reach statistical significance due to the small sample size. In the patients with uncontrolled disease, one patient had an unchanged AIGA titer after antibiotic treatment [34]. Three patients had reached a 50 ~ 75% reduction after CTX, although their AIGA titer still remained as high as 1:50,000 [28]. In the patients with controlled disease, the AIGA titer was reduced by 25 ~ 99.5% after antibiotic treatment with or without RTX and CTX treatment.

**Table 1:** Geographic and clinical characteristics of the 195 patients enrolled in the study

	Total (*n* = 195)	Controlled (*n* = 119)	Uncontrolled (*n* = 76)	*P*-value
Age (years) (range)	55 (10 ~ 87)	57 (10 ~ 87)	51 (20 ~ 81)	0.233
Male (%)	101 (51.8%)	64 (53.8%)	37 (48.7%)	0.557
Comorbidity (%)	61 (31.3%)	31 (26.1%)	30 (39.5%)	0.058
Diagnostic time (months) (range)	12 (0.7 ~ 612)	11 (0.7 ~ 120)	11 (1 ~ 612)	0.569
Disease time (months) (range)	28 (1.6 ~ 279)	28 (3.5 ~ 156)	28 (1.6 ~ 279)	0.878
No. of infection sites (range)	3 (1 ~ 8)	3 (1 ~ 7)	2 (1 ~ 8)	0.028
Constitutional symptoms (%)	138 (70.8%)	89 (74.8%)	49 (64.5%)	0.147
Skin involvement (%)	71 (36.6%)	52 (44.1%)	19 (25.0%)	0.009
Abscess formation (%)	44 (22.6%)	29 (24.4%)	15 (19.7%)	0.487
Bone infection (%)	90 (46.4%)	59 (50.0%)	31 (40.8%)	0.239
Lung infection (%)	131 (67.5%)	75 (63.6%)	56 (73.7%)	0.159
Lymphadenopathy (%)	145 (74.4%)	94 (79.0%)	51 (67.1%)	0.067
Bloodstream infection (%)	43 (22.2%)	27 (22.9%)	16 (21.1%)	0.860
Bone marrow infection (%)	20 (10.3%)	15 (12.7%)	5 (6.6%)	0.228
No. of species (range)	2 (1 ~ 6)	1 (1 ~ 6)	2 (1 ~ 5)	0.032
NTM infection (%)	151 (77.4%)	107 (89.9%)	44 (57.9%)	< 0.001
TM infection (%)	58 (29.7%)	21 (17.6%)	37 (48.7%)	< 0.001
No. of episodes (range)	2 (1 ~ 9)	1 (1 ~ 8)	2 (1 ~ 9)	< 0.001
Recurrent infections (%)	79 (56.0%)	37 (43.5%)	42 (75.0%)	< 0.001
No. of Abx usage (range)	4 (1 ~ 18)	4 (1 ~ 12)	4 (1 ~ 18)	0.480
Total treatment time (months) (range)	17 (0.5 ~ 156)	18 (0.67 ~ 156)	11.15 (0.5 ~ 67)	0.185
Follow-up time (months) (range)	16 (0.3 ~ 96)	16.5 (0.3 ~ 96)	15.5 (0.6 ~ 72)	0.984
RTX treatment (%)*	38 (19.5%)	29 (24.4%)	5 (6.6%)	0.002
CTX treatment (%)^&^	21 (10.8%)	15 (12.6%)	5 (6.7%)	0.230

No. of pathogen: total number of infection pathogens during disease course. *NTM*, nontubercular mycobacterium; *TM*, Talaromyces marneffei; No. of episode: number of infection episodes during disease course. No. of Abx usage: the total number of antibiotic used during disease course. *RTX*, rituximab; *CTX*, cyclophosphamide. *3 patients received RTX but did not report its effect and 1 patient received RTX before the diagnosis of the disease; these four patients were therefore excluded in outcome study. ^**&**^1 patient received CTX before the diagnosis of the disease and was excluded in outcome study

In the crude logistic analysis, skin involvement, comorbidity, NTM infection, TM infection, RTX treatment, and recurrent infections were all significantly associated with disease outcome (Table 2). In the multivariate logistic analysis, skin involvement (OR = 3.25, 95% CI 1.187 ~ 8.909, *P* value = 0.022), NTM infection (OR = 4.74, 95% CI 1.300 ~ 17.30, *P* value = 0.018), and recurrent infections (OR = 0.22, 95% CI 0.086 ~ 0.551, *P* value = 0.001) remained significantly associated with prognosis.

**Table 2:** Single and multivariate logistic analysis for disease control

	OR	*P*-value	95% CI
Crude analysis			
Comorbidity	0.54	0.050	0.290 ~ 0.999
Skin involvement	2.36	0.008	1.254 ~ 4.455
NTM infection	6.48	< 0.001	3.061 ~ 13.74
TM infection	0.24	< 0.001	0.125 ~ 0.456
No. of episode	0.59	< 0.001	0.448 ~ 0.784
Recurrent infection	0.26	< 0.001	0.122 ~ 0.539
RTX treatment	4.57	0.003	1.685 ~ 12.42
Multivariate logistic analysis			
Comorbidity	1.18	0.728	0.468 ~ 2.968
Skin involvement	3.25	0.022	1.187 ~ 8.909
NTM infection	4.74	0.018	1.300 ~ 17.30
TM infection	0.75	0.644	0.218 ~ 2.563
Recurrent infection	0.22	0.001	0.086 ~ 0.551
RTX treatment	3.67	0.071	0.895 ~ 15.07

*NTM*, nontubercular mycobacterium; *TM*, Talaromyces marneffei; No. of episode: number of infection episodes during disease course. *RTX*, rituximab; *CTX*, cyclophosphamide

## Discussion

AIGA-associated adult onset immunodeficiency (AOID) is a rare disease that has only been reported in recent years. In an effort to promote treatment and prognosis, we analyzed 195 patients with reported treatment protocols and outcomes. The clinical phenotype was consistent with previous reports [35]. The disease imposes a significant burden on medical centers, with 195 patients bearing 362 pathogens. The patients suffered from disseminated infections, a prolonged disease course (median 28 months) and a high rate of recurrence (56.0%). A median of 4 antibiotics were used with a median of 17 months of treatment, while the effective rate was only 40.5% (79/195). Long-term antibiotic treatment can lead to other complications, such as liver and renal toxicity. One author reported one AOID patient with posterior corneal deposits after long-term treatment with rifabutin [36]. Thus, this disease urged high clinical awareness.

The detection of the antibody was crucial for the diagnosis of the disease. However, the median diagnostic time was 12 months. Although diagnostic time did not impact the disease outcome in the present study, several cases have reported delayed diagnosis leading to missed treatment opportunity. Thus, the detection of the disease should be improved. As an emerging infectious disease, not every medical center have the ability to run tests to detect the antibody. Some authors have suggested using the QuantiFERON Gold In-tube assay to screen the autoantibody [37]. Nevertheless, as the disease prevails in Southeast Asia, clinicians should be alerted about the disease in patients with these ethical background and who are presenting with opportunistic infections.

In the present review, skin involvement was significantly associated with disease control. Skin involvement was common in AOID patients, with both reactive lesions and infective skin lesions [38]. As dermatologically reactive lesions usually suggest infections of other sites, skin involvement might alert investigation and therefore management. On the other hand, recurrent infection was significantly associated with uncontrolled disease, as shown in the multivariate logistic analysis (OR = 0.22, 95% CI 0.086 ~ 0.551, *P* value = 0.001). Similarly, in one Chinese cohort, patients with positive AIGA had more coinfections, multiple infection sites, and increased inflammatory markers [39]. In a previous Thai cohort, compared with patients with stable disease (*n* = 32), patients with active disease (*n* = 31) had significantly lower hematocrit, higher white cell count, higher C reactive protein (CRP), higher erythrocyte sedimentation rate, and higher AIGA titer, with CRP showing the highest area under the receiver operating characteristic (ROC) curve (AUC) of 0.88 (95% CI 0.81–0.95) [16]. These results suggested that although more clinical symptoms might urge more clinical attention, severe and recurrent infections were worse prognostic factors of the disease outcome.

NTM was associated with better disease outcome, probably due to the high awareness of the disease. To our notice, *T. marneffei* (TM) infection was associated with poor disease control in the crude analysis. TM was the second most common pathogen in our review, probably due to its high prevalence in Southeast Asia. In a recent cohort in South China, 55/58(95%) of patients with non-HIV-infected TM infection had AIGAs [40]. In two other Chinese cohorts with disseminated TM infections, patients with high AIGA titers tended to have persistent TM infection, higher rates of recurrence, worse treatment outcomes, and higher mortality rates [41, 42]. Although TM infection did not reach significance in the multivariate logistic analysis due to the small sample size, as TM prevalence coincides with AOID prevalence, AIGA associated TM infection should receive more clinical attention and even advanced treatment.

Due to the small number, we did not include the AIGA titer in the outcome analysis, although an observation study showed that patients with controlled disease had a significant AIGA titer decline, while patients with uncontrolled disease still had a high AIGA titer after treatment. In the study by Hong et al. [8], the antibody titer was higher in the patients with active infections, and the odds of having an infection increased 2.98 ~ 10.85 times with a 1-log increase in autoantibody titer (*p* < 0.01). Further functional study showed that with improvement of the neutralizing capacity, the patient’s plasma showed partial reconstitution of IFNγ induced chemokine expression [4], and the STAT1-PI had significantly increased (2.3 ± 4.7% to 16.1 ± 33.8%, *p* < 0.01), though still only 16.1% to the normal subjects [43]. As even partial improvement in IFNγ signaling could effectively improve the control of opportunistic infections [44], autoantibody suppression and IFNγ signaling restorations have been explored in recent years.

RTX, a specific CD20 + B cell antibody, has been reported in AOID patients with progressive infections, which achieved 35.2% AIGA reduction and 73.5% efficiency in the present review. CTX, a general lymphocyte suppressor, was also reported in several Asian countries due to the unavailability of RTX. In the present review, CTX treatment achieved 96.25% AIGA reduction and 75% efficiency. Both of them were reported to be higher in patients with controlled disease, although not reaching statistical significance due to the small sample size.

Both of them were reported to be higher in patients with controlled disease, although not reaching statistical significance due to the small sample size. In a Japanese cohort with 13 patients, the antibody titer decreased from 1274 ± 2682 E.U. to 184 ± 206 E.U. (*P* < 0.05) after immunosuppressant therapy [43]. In a Thai cohort with 17 patients, 6 of whom received RTX and 11 received CTX due to progressive infections, the effective rates of RTX and CTX were comparable to each other (4/6 and 8/11, respectively) [21].

However, it should be noted that there were patients who failed both RTX and CTX [21], and the patients recurred after RTX cessation [4, 45]. In the present study, RTX achieved only 35.2% AIGA reduction. RTX is a specific CD20 + B cell antibody, which has no direct effect on mature, long-lived plasma cells. CTX is a general lymphocyte inhibitor, which might explain the greater AIGA reduction. Bortezomib, a proteasome inhibitor targeting plasma cells, has shown further suppression of the autoantibodies after RTX failure [22]. Daratumumab, a CD38 + plasmablast and plasma cell antibody, further reduced tissue plasma cells, total IgG levels, AIGA titers and disease progression [27]. As bortezomib and daratumumab have shown successful usage in refractory autoimmune disease, these two agents could be considered in AOID patients with progressive disease. On the other hand, Lin et al. [3] generated an epitope-erased IFNγ (EE-IFNγ) that lowerd the binding affinity of AIGA by approximately 40%. In an ex vivo experiment, EE-IFNγ reactivated the IFNγ downstream pSTAT1signaling and IL-12 production. Thus, this EE-IFNγ might be a promising way to overcome the autoantibody and restore the immune system.

The present study had some limitations. First, the case number was small due to the rarity of the disease, and the data had high heterogeneity due to the variety of the disease itself, both of which might result in systemic bias. Second, this was only a semiquantitative study based on previous case reports, limiting further clinical investigation. Furthermore, patients without progressive infections but still under treatment were included in the controlled group due to the high recurrence rate; as a result, more efforts were required to definitely control the disease. Finally, real-world clinical management is much more complicated, requiring constant adjustment of treatment. Patients’ tolerance, compliance, preference, and medical availability might profoundly affect treatment choice in different healthcare settings. Nevertheless, the results from this review might still provide valuable information for clinical management and further investigation of the disease.

In conclusion, we have summarized previous reports of anti-IFNγ antibody associated immunodeficiency and found that skin involvement, NTM infection, and recurrent infections might be associated with disease outcome. Anti-IFNγ antibody titer suppression and immune system restoration are important for controlling the disease. Patients with diffusion opportunistic infection might benefit from screening of the AIGA, especially in those without other immunodeficient conditions and with Asian backgrounds. The treatment protocols should be tailored in each patient according to their infection, autoantibody titer, and treatment response, whereas large multicenter cohort studies are prudent to establish standard treatment protocols for the disease and to improve disease outcome.

## Supplementary Information

Below is the link to the electronic supplementary material.

Supplementary file1 (XLSX 41 KB)

## Data Availability

All data are available from the corresponding authors upon reasonable request (contact via huangxiaoming@pumch.cn and zxjpumch@126.com).
